# HIV and hepatitis B, C co-infection and correlates of HIV infection among men who have sex with men in Rwanda, 2021: a respondent-driven sampling, cross-sectional study

**DOI:** 10.1186/s12879-024-09206-2

**Published:** 2024-03-23

**Authors:** Eric Remera, Elysee Tuyishime, Catherine Kayitesi, Samuel S. Malamba, Beata Sangwayire, Justine Umutesi, Horacio Ruisenor-Escudero, Tom Oluoch

**Affiliations:** 1 Division of Global HIV and TB, Global Health Center (GHC), US Centers for Disease Control and Prevention (CDC), Kigali, Rwanda; 2 Institute of HIV Disease Prevention and Control, Rwanda Biomedical Centre (RBC) ), Kigali, Rwanda; 3 Division of Global HIV and TB, Global Health Center (GHC), US Centers for Disease Control and Prevention (CDC), Atlanta, GA USA

**Keywords:** HIV, MSM, Recency, SSA, Rwanda, RDS, Coinfection, Hepatitis B, Hepatitis C

## Abstract

**Background:**

Men who have sex with men (MSM) are a key population group disproportionately affected by HIV and other sexually transmitted infections (STIs) worldwide. In Rwanda, the HIV epidemic remains a significant public health concern, and understanding the burden of HIV and hepatitis B and C coinfections among MSM is crucial for designing effective prevention and control strategies. This study aims to determine the prevalence of HIV, hepatitis B, and hepatitis C infections among MSM in Rwanda and identify correlates associated with HIV infection within this population.

**Methods:**

We used respondent-driven sampling (RDS) to recruit participants between November and December 2021. A face-to-face, structured questionnaire was administered. Testing for HIV infection followed the national algorithm using two rapid tests: Alere Combo and STAT PAK as the first and second screening tests, respectively. Hepatitis B surface antigen (HBsAg) and anti-HCV tests were performed. All statistics were adjusted for RDS design, and a multivariable logistic regression model was constructed to identify factors associated with HIV infection.

**Results:**

The prevalence of HIV among MSM was 6·9% (95% CI: 5·5–8·6), and among HIV-positive MSM, 12·9% (95% CI: 5·5–27·3) were recently infected. The prevalence of hepatitis B and C was 4·2% (95% CI: 3·0–5·7) and 0·7% (95% CI: 0·4–1·2), respectively. HIV and hepatitis B virus coinfection was 0·5% (95% CI: 0·2–1·1), whereas HIV and hepatitis C coinfection was 0·1% (95% CI: 0·0–0·5), and no coinfection for all three viruses was observed. MSM groups with an increased risk of HIV infection included those who ever suffered violence or abuse because of having sex with other men (AOR: 3·42; 95% CI: 1·87–6·25), those who refused to answer the question asking about ‘ever been paid money, goods, or services for sex’ (AOR: 10·4; 95% CI: 3·30–32·84), and those not consistently using condoms (AOR: 3·15; 95% CI: 1·31–7·60).

**Conclusion:**

The findings suggest more targeted prevention and treatment approaches and underscore the importance of addressing structural and behavioral factors contributing to HIV vulnerability, setting interventions to reduce violence and abuse against MSM, promoting safe and consensual sexual practices, and expanding access to HIV prevention tools such as condoms and preexposure prophylaxis (PrEP).

## Introduction

Several countries are approaching sustained HIV epidemic control as defined by the U.S. President’s Emergency Plan for AIDS Relief (PEPFAR) [1]. In this context, the Joint United Nations Programme on HIV/AIDS (UNAIDS) calls for setting ambitious targets for reaching 95% of all people living with HIV to know their HIV status, 95% of all people with HIV infection to receive sustained antiretroviral therapy, and 95% of all people receiving antiretroviral therapy to have viral suppression by 2025, as well as the 2030 target of ending AIDS as a public health threat [2, 3]. To achieve this target, it would benefit from adjusting existing general population approaches, leveraging more targeted interventions for key HIV-affected population subgroups, and addressing equity across relevant population subgroups.

In 2021, key HIV population (KP) groups, including female sex workers (FSW), gay men and other men who have sex with men (MSM), people who inject drugs (PWID), and transgender people (TG) and their sexual partners, accounted for 70% of HIV infections globally [4]. HIV key populations account for 51% of new HIV infections, and the risk of acquiring HIV was 28 times higher among MSM than other adult men in sub-Saharan Africa [4]. A renewed focus on the HIV epidemic in sub-Saharan Africa has brought attention to the importance of key affected populations. With this renewed approach, the Rwanda HIV Population Research Technical Working Group recommends the regular conduct of biobehavioral surveillance among KPs to timely monitor HIV epidemics among KPs [5].

Research among MSM in sub-Saharan Africa has reported HIV prevalence rates that are usually higher than the national prevalence among the general population. These adjusted HIV prevalence rates range from 27·2% in Swaziland to 13·5% in Kampala, Uganda, and as high as 34·9% in Abuja, Nigeria, and 44·4% in Yaoundé, Cameroon [6–9]. Many studies have reported a high prevalence of frequent unprotected anal intercourse, which is particularly concerning given the low median age of participants 24 (IQR: 22–28) and suggests that if immediate HIV prevention efforts designed and focused specifically on MSM are not implemented, HIV prevalence might continue to rise [10–14].

Furthermore, hepatitis B virus (HBV) and hepatitis C virus (HCV) infection are significant public health concerns, especially among high-risk groups for HIV infection, due to increases in morbidity and care costs once one is coinfected with HIV [15–17]. In Rwanda, the overall prevalence of acute or chronic hepatitis B among adults aged 15–64 years was 2·0%, whereas that of hepatitis C among adults in Rwanda was 1·2% [18]. Studies have consistently shown that MSM have a higher prevalence of HBV and HCV infection compared to the general population [19–21]. Due to shared routes of transmission, HIV coinfection with other sexually transmitted infections (STIs) is relatively common and can increase susceptibility to HBV and HCV infection, posing several challenges in treatment and management [22, 23].

Rwanda has made remarkable progress toward scaling up access to HIV prevention and care [24]. The country adopted new treatment guidelines in 2016 that include immediate treatment for all people living with HIV. Research that identifies gaps in the HIV continuum of care has helped policymakers design a responsive health system that has significantly decreased mortality and improved life expectancy [25, 26]. However, in 2018, biobehavioral surveillance of MSM in the capital city of Rwanda indicated that countrywide, MSM might be more likely to experience a higher burden of HIV due to a concentrated HIV prevalence [27]. Furthermore, the biobehavioral surveillance of FSWs found an alarmingly high HIV prevalence of 51%, in contrast to a national prevalence of 3% [25, 28].

The national guidelines for HBV, HCV, and STIs management in Rwanda highlight MSM among groups with an increased risk of HBV and HCV infections. The guidelines sate that the diagnosis of HBV infection is based on the presence of HBsAg, which confirms the chronicity if the person is already HIV-positive. Patients who are HBsAg negative directly start the HBV vaccine series. All HIV-HBV-infected patients should start treatment for both infections as per the HIV “treat all” guidelines of 2016 up to now. In addition, HIV treatment includes an HBV-active agent such as TDF/TAF or entecavir, and the recommended treatment is for life.

Furthermore, the guidelines recommend a two-step HCV diagnostic process: firstly, performing serological testing by conducting anti-HCV antibody testing, followed by confirmation of the current infection by conducting viral load testing for anti-HCV positivity. For HCV management, the guidelines recommend treating all patients, both adults and children aged 3 years and older, weighing at least 14 kg and above, with detectable HCV RNA viral load should be initiated on treatment with direct acting antivirals (DAAs), except for pregnant women.

In Rwanda, understanding the prevalence of HIV, HBV, and HCV infection among MSM is crucial for designing targeted prevention and treatment interventions. This study aimed to determine the prevalence of HIV, HBV, and HCV infection among MSM in Rwanda and identify potential correlates of HIV infection within this population.

## Methods

### Study design and population

Data came from a cross-sectional, biobehavioral survey (BBS) of MSM in Rwanda. Respondent-driven sampling (RDS) was used as a sampling approach to recruit MSM between November and December 2021 [29]. Seeds were identified through community leaders and associations across the country and were screened by study staff to ensure they had attributes necessary to facilitate productive recruitment. Selection criteria included large social network sizes, respected members of their communities, and motivation to contribute to the achievement of the study objectives by encouraging their peers to participate.

Each seed was given three RDS coupons to recruit peers from their networks who were members of the target population and who they knew and who knew them. Recruitment continued by providing each recruit with three RDS coupons until the target sample size was reached. Eligibility criteria for participation included being a male at birth, at least 18 years of age, residing in Rwanda for at least 12 months, having reported having had anal or oral sex with a man in the last 12 months, and consenting to participate in the study.

**Fig. 1:**
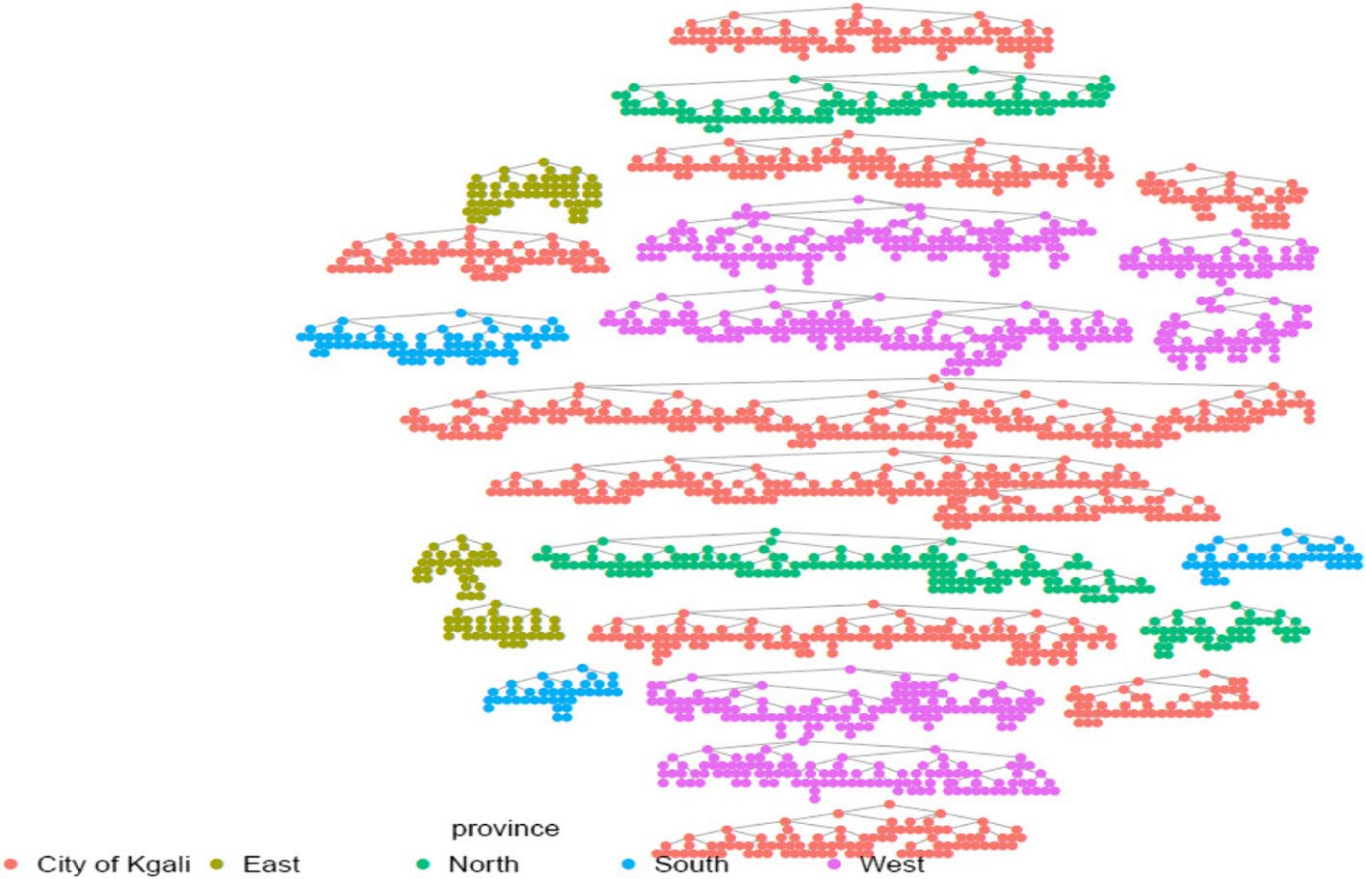
Recruitment chains for each RDS seed, Rwanda, 2021

There were eight study sites distributed according to the administrative provinces, and three seeds were selected at each study site, making 24 seeds in total. Figure 1 shows the recruitment chains generated by each of the 24 RDS seeds. Compensation of 3,000 Rwandan francs (RWF) corresponding to 3·6 United States dollars (USD) with 2021 as the base year to cover transport expenses and an additional 2,000 RWF (2·4 USD) to compensate for time spent was provided. Secondary compensation of 3,000 RWF (3·6 USD) for each eligible recruit was offered, and participants returned to the study office approximately two weeks after their study visit to collect their recruitment reimbursement.

### Data collection and measures

**Fig. 2:**
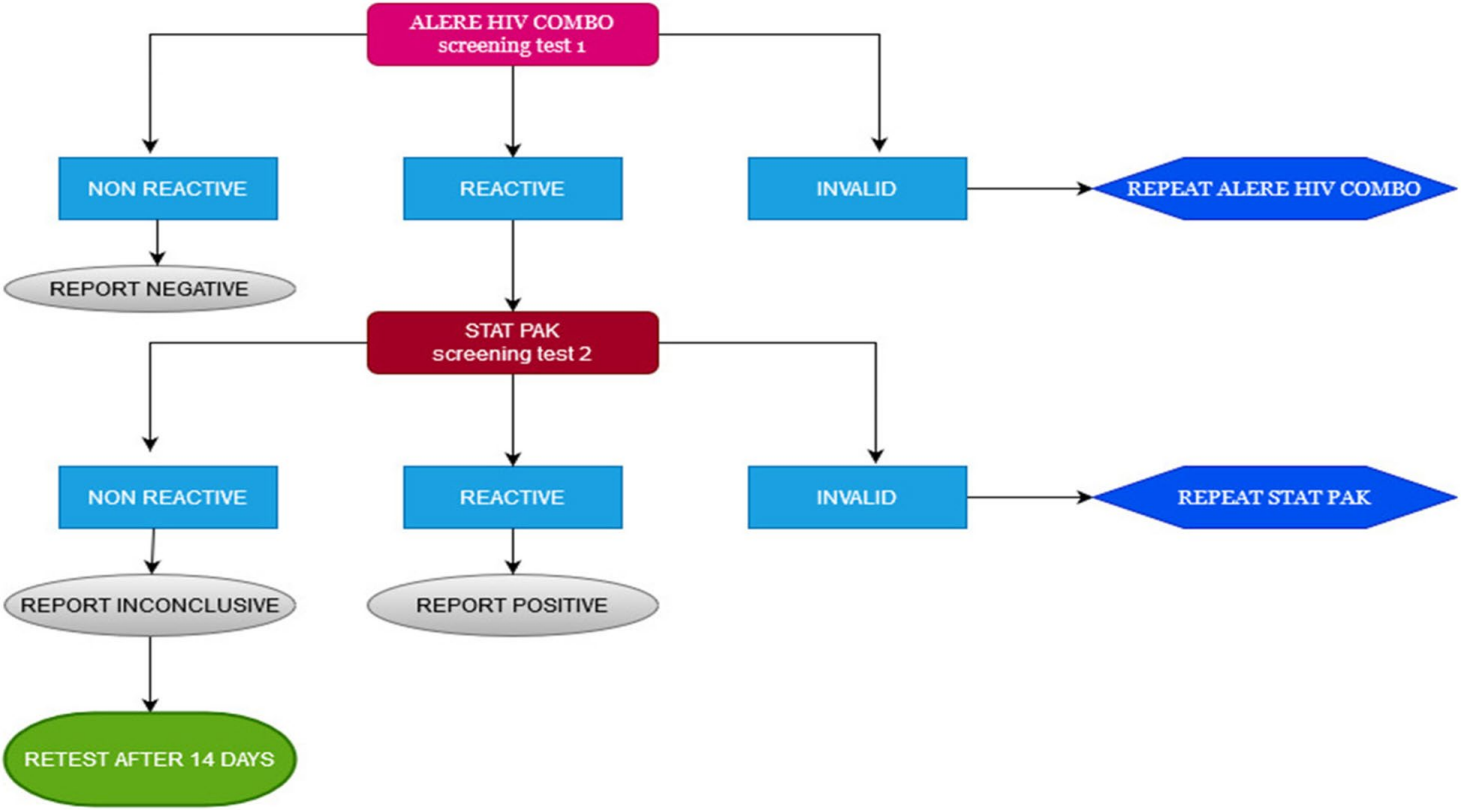
HIV Rapid Test Algorithm, National HIV guideline, Rwanda 2018

**Fig. 3:**
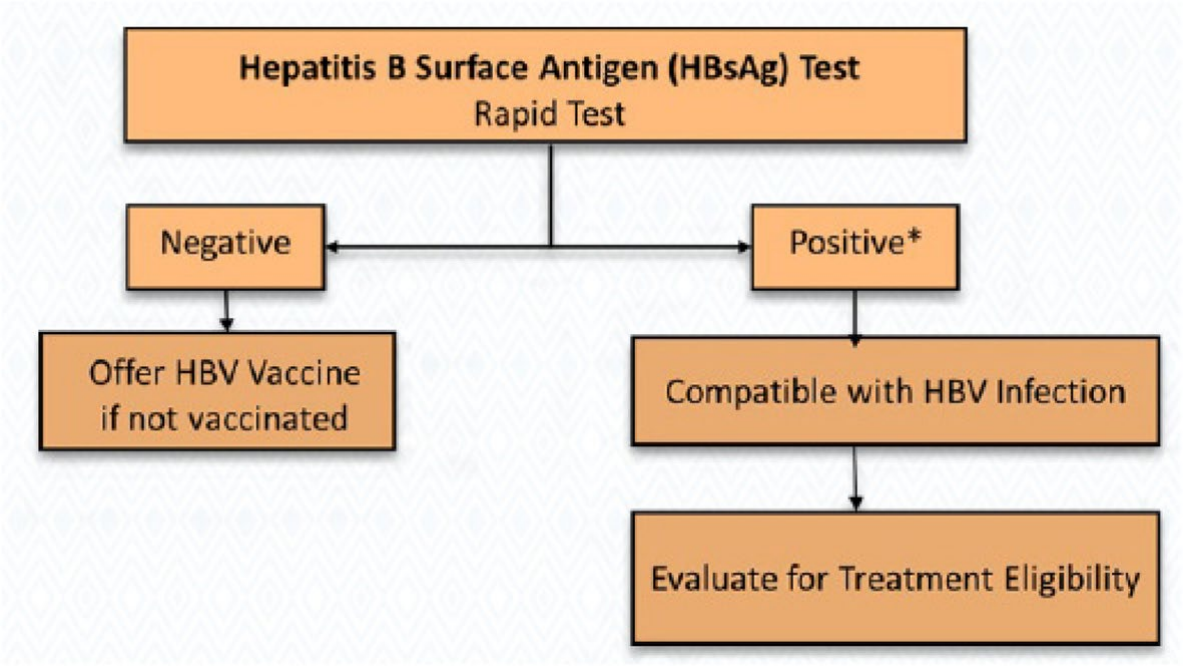
Algorithm for screening and diagnosis of chronic HBV infection, National Viral Hepatitis and STIs guideline, Rwanda 2019

**Fig. 4:**
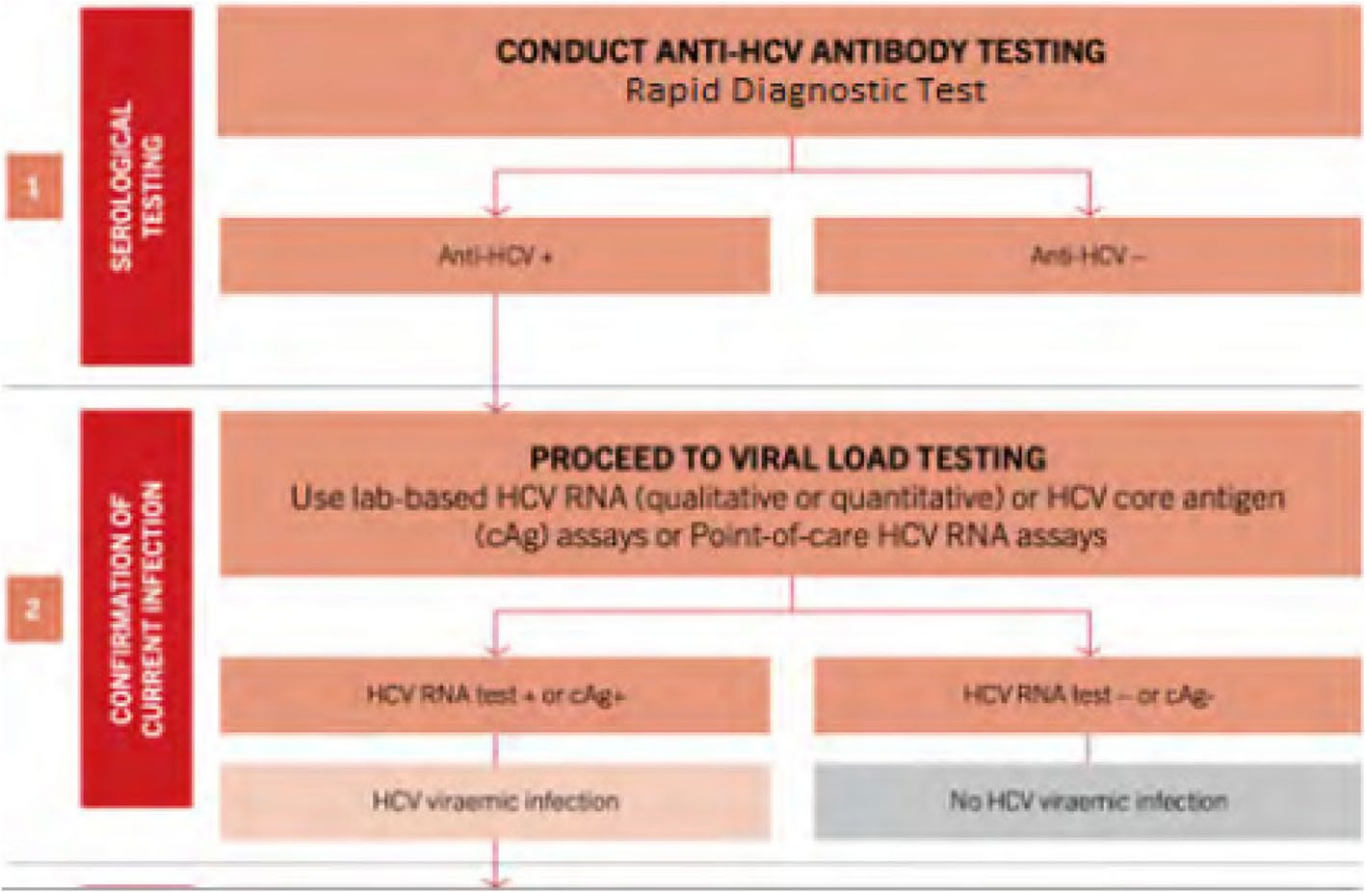
Algorithm for screening and diagnosis of HCV infection, National Viral Hepatitis and STIs guideline, Rwanda 2019

The survey was interviewer-administered, and data were collected on Android tablets using a standardized, structured questionnaire. The questionnaire collected data on demographics, sexual risk behaviors, HIV and STI knowledge, attitudes and beliefs, and other health-related information. Following pretest counseling, HIV tests were performed on all consenting participants by following the national-approved HIV testing algorithm of two tests: Alere Combo as the first screening test and Stat Pak as the second screening test (Fig. 2). Using the Rapid Test for Recent Infection (RTRI) assay and viral load testing, all participants diagnosed as HIV-positive were classified as likely having acquired an HIV infection within the last 12 months (i.e., recent infection) or having acquired an HIV infection more than a year ago (i.e., long-term infection). All HIV-positive participants were referred to health facilities for treatment after posttest counseling. Rapid hepatitis B (Fig. 3) and C (Fig. 4) tests were conducted on all participants to detect hepatitis B surface antigen (HBsAg) and anti-HCV using rapid diagnostic tests (RDTs), respectively.

### Data analysis

The individual-level survey data were extracted directly from the Open Data Kit (ODK) and imported into RDS-Analyst software to be cleaned and recoded prior to any analysis. Since crossover recruitment between study sites was not observed, site-level data were analyzed separately, which means that there were eight RDS surveys where weight calculations considered the site-corresponding MSM population size estimates (PSE) [30].

Study sites were distributed by province: North with MSM PSE of 2,375, South with PSE of 2,109, and East with PSE of 2,287 had each one study site, whereas the City of Kigali with PSE of 7,842 and West with PSE of 2,469 had each 3 and 2 study sites, respectively, based on the MSM PSE 2023 published results [30]. To obtain weights for each study site, the provincial-level PSEs for the city of Kigali and Western Province were split to obtain site-level PSEs to be used for site-level weight computation.

As a result, the PSEs for the three study sites from the city of Kigali were assigned as follows: Gatenga HC with a PSE of 2,300; Remera HC with a PSE of 2,300; and We-ACT with a PSE of 3,200. Site-level MSM PSEs for the two sites from the West Province were assigned to Gisenyi HC with a PSE of 1,200 and Gihundwe HC with a PSE of 1,300. All site-level PSEs within the city of Kigali and West Province were assigned, assuming a distribution of site-level MSM PSEs to follow urban/rural KP dynamics (higher in urban areas and less in rural areas) using urban/rural ratios from the 2012 population census.

Finally, to obtain the nationwide pooled estimates, all study sites were aggregated using the “Aggregate Estimate’ option available within RDS-Analyst using site-level PSEs for all 8 study sites across the country [30]. Analyses were conducted using RDS-Analyst, software developed for the analysis of complex RDS data [31]. The data were later exported into a standard statistical package (STATA version 17 (College Station, TX)) with individual weights for further analyses [32]. Analyses provide adjusted population point estimates and a 95% Confidence Intervals (95%CI) of key indicators of interest.

**Fig. 5:**
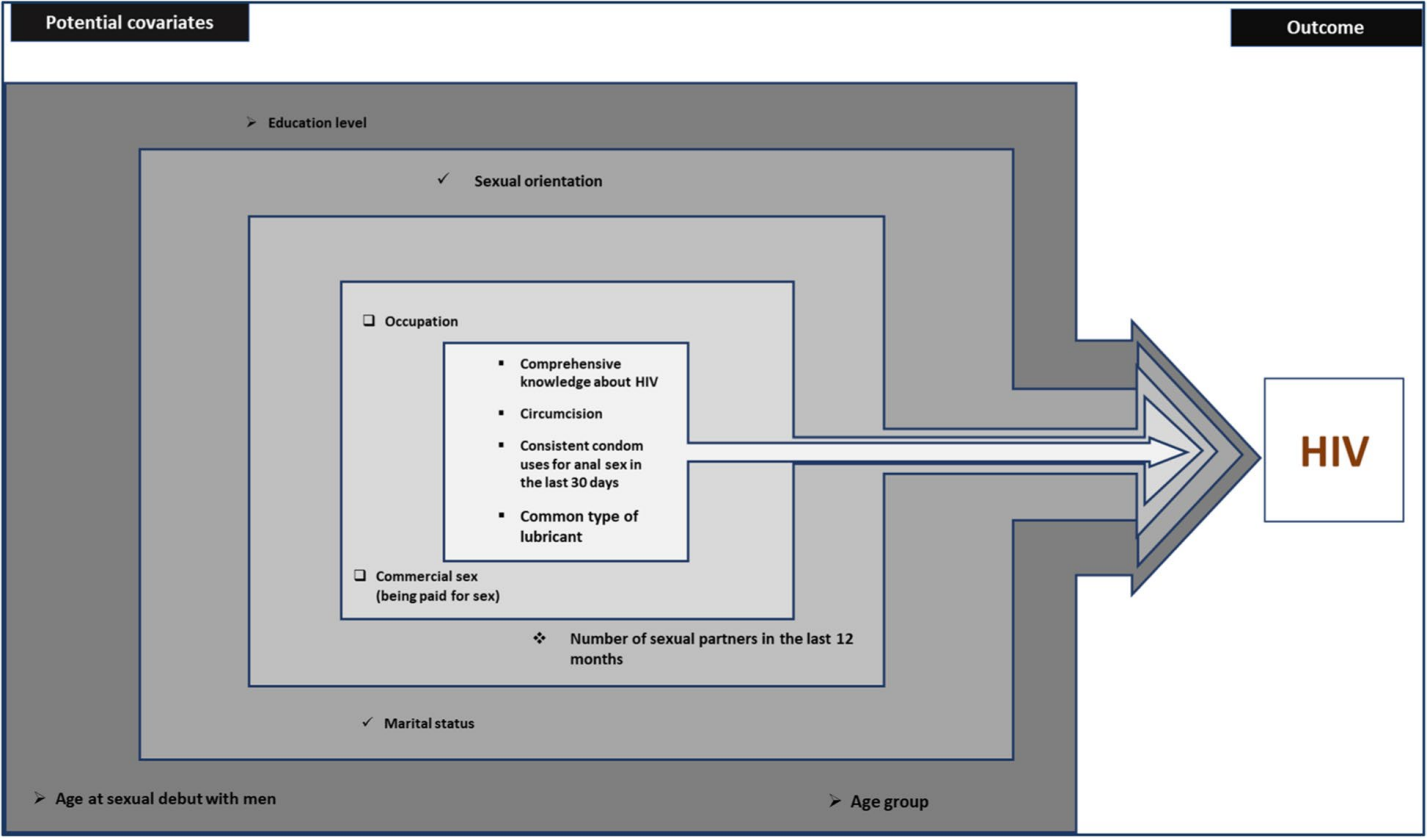
Conceptual framework for potential covariates of HIV infection among MSM, Rwanda, 2021

Data were summarized using descriptive statistics as either proportions or medians and associated interquartile ranges (IQRs) for continuous variables. All statistics were weighted according to RDS-generated weighting techniques using RDS-analyst software. The primary outcome variable was summarized as the proportion of the number of participants with a positive HIV diagnosis divided by the total number of participants tested for HIV. A review of the literature identified factors associated with HIV infection among MSM in the region and the strength of the association between each of these variables and the primary outcome variable at both the bivariable and multivariable levels (Fig. 5). We constructed a bivariate logistic regression model for each individual factor to observe individual factor associations with the outcome of interest. For all significant factors at the bivariate logistic regression level, they were considered one by one for the multivariable logistic regression model. An explanatory, multivariable logistic regression model was constructed using forward stepwise selection of variables that minimized the Akaike information criterion (AIC) and model testing using the likelihood ratio test (Fig. 5). Effect estimates are presented as odds ratios (ORs) and adjusted odds ratios (AORs) along with associated 95% confidence intervals (95% CIs). All statistical tests were two-sided with alpha set at 0.05.

### Ethical considerations

The survey received ethical approval from the local Institutional Review Board and the Rwanda National Ethics Committee (RNEC). It was also reviewed in accordance with the US. Centers for Disease Control and Prevention (CDC) human research protection procedures and was determined to be research with CDC nonengaged. Since same-sex relationships remain stigmatized in Rwanda, we anticipated possible risks of physical and/or verbal violence in cases where a study participant was identified in the community. Therefore, the study investigator ensured that the study was conducted anonymously to protect the identity of participants and ensure confidentiality of the data collected; no names or any other personally identifiable information were recorded anywhere.

Completed questionnaires (identifying individuals by only identification numbers) were kept with the study coordinator during the fieldwork. All forms containing any study information were kept in a locked cabinet accessible to only authorized study personnel. Additionally, the electronic data set identified by the unique code was password-protected and accessed by authorized personnel using a computer backed up to a server located at the Rwanda Biomedical Center. Furthermore, for all study members participating in human subjects’ research and data collection, it was a requirement for the study team to have participated in training on human subjects’ research, confidentiality, and interviewing techniques before commencing study activities. A confidentiality agreement was signed by all study investigators, coinvestigators, and data collectors.

### Role of funding source

The funders of the study had no role in the study design, in the collection, analysis, and interpretation of data, in the writing of the report, or in the decision to submit the paper for publication.

In total, 3,094 coupons were distributed. Of these coupons, 2,304 were returned by potential MSMs to the study site and were valid, corresponding to a coupon return rate of 74·5%. Of the 2,304 potential participants, 66 did not have valid coupons or were ineligible to participate. Therefore, the total number eligible was 2,238, with 2,211 (98·8%) consenting to providing interviews and blood samples for all survey biomarkers.

## Results

Almost half of the MSM population was (49·9%) less than 25 years old, and those aged 25–29 years represented 25·7%. The median age was 25 (IQR: 22–29), and 24·4% were more than 30 years old. More than two in five MSM reside in the city of Kigali (45·7%). Most MSM were single (90·0%), with 46·8% having not gone beyond primary school, and almost one in five were unemployed (19·5%). Lifetime drug use among MSM was 14·5%, with 4·7% taking drugs just before having sex and 1·1% having ever injected drugs. Slightly less than half of the MSM population (43·2%) had their first anal sex with men while they were under 18 years old. Slightly more than a third of the MSM (37·6%) reported consistent condom use for anal sex. The majority of MSM reported being circumcised (89·1%), with a median age of 12 (IQR: 4–18) years at circumcision. Finally, the median number of sexual partners reported in the 12 months prior to being surveyed was 3 (IQR: 2–5) (Table 1).

**Table 1:** Descriptive statistics of the sample of MSM with RDS-adjusted proportions (*n* = 2,211), Rwanda, 2021

	**Frequency**	**RDS Adjusted %**	**95% CI**
**Age group (years)**			
18–19	181	8·5	[7·0—10·3]
20–24	840	41·4	[38·4—44·5]
25–29	573	25·7	[23·1—28·4]
30 +	615	24·4	[21·9—27·0]
**Province**			
East	126	13·4	[11·1—16·1]
Kigali	1,021	45·7	[42·7—48·8]
North	303	14·0	[11·9—16·3]
South	152	12·4	[10·3—14·9]
West	609	14·5	[13·1—16·1]
**Level of education completed**			
No education/primary	962	46·8	[43·6—49·9]
Vocational/secondary/higher	1,157	53·2	[50·1—56·4]
**Main occupation**			
Not employed	449	19·5	[17·2—22·1]
Long-term regular work	422	17·4	[15·2—19·8]
Students	206	11·1	[9·3—13·2]
Parttime staff/Casual laborer	549	24·8	[22·2—27·5]
Other	585	27·2	[24·6—30·0]
**Current marital status**			
Single	1,993	90·0	[87·9—91·8]
Ever married	217	9·9	[8·1—12·1]
Refused to answer	1	0·1	[0·0—0·5]
**Ever took drugs for any reason**			
Yes	365	14·5	[12·6—16·6]
No	1,846	85·5	[83·4—87·4]
**Ever took drugs just before having sex**			
Yes	142	4·7	[3·6—6·1]
No	2,004	95·1	[93·7—96·2]
Refuse to answer	5	0·2	[0·1—0·5]
**Ever injected drugs**			
Yes	35	1·1	[0·6—2·1]
No	2,175	98·9	[97·9—99·4]
Refuse to answer	1	0·0	[0·0—0·1]
**Age at first sexual intercourse with a man (years)**			
< 15	143	4·8	[3·8—6·1]
15–18	832	38·4	[35·4—41·5]
19–22	694	35·7	[32·7—38·7]
23–25	251	11·7	[9·8—13·9]
26 +	219	9·4	[7·8—11·2]
**Circumcised**			
*Yes*	1,971	89·1	[87·1—90·8]
*No*	238	10·8	[9·1—12·9]
*Refuse to answer*	2	0·1	[0·0—0·2]
**median age at circumcision [IQR]**	1,971	12 Years	[4–18]
**Median number of sexual partners reported in the 12 months prior the survey [IQR]**	2,211	3	[2–5]
**Ever suffer any violence or abuse because of having sex with other men**			
Yes	237	10·3	[8·6—12·3]
No	1,972	89·7	[87·6—91·4]
Refuse to answer	2	0·0	[0·0—0·2]
**Ever been paid money, goods, or services for sex**			
Yes	580	22·6	[20·2—25·2]
No	1,555	75·7	[73·0—78·2]
Refuse to answer	16	1·7	[0·9—3·2]
**Condom use with all partners (general)**			
Consistent	720	37·6	[34·6—40·7]
Not consistent	1,404	61·1	[58·0—64·1]
Refused to Answer	27	1·3	[0·8—2·2]
**Frequency of alcohol consumption over the last 30 days**			
Every day	168	7·5	[6·0—9·3]
About every day	532	32·5	[29·2—35·9]
At least once a week	769	50·5	[46·9—54·1]
At least once a month	134	8·4	[6·6—10·7]
None	11	0·8	[0·4—1·8]
Do not know	3	0·3	[0·1—1·1]

*RDS* Respondent-driven Sampling, *IQR* Inter Quartile Range

The adjusted RDS HIV prevalence among MSM was found to be 6·9% (95% CI: 5·5–8·6), and the highest HIV prevalence was in the Eastern province with 10·3% (CI: 5·4–18·7), followed by the City of Kigali with 7·6% (CI: 5·7–10·1). The HIV prevalence in other provinces ranged from 4·5% to 5·3%. The prevalence of HIV RITA recent (HIV infection that occurred in the last 12 months) in MSM was 12·9% (95% CI: 5·5–27·3) among those MSM diagnosed with HIV during the survey. The prevalence of hepatitis B and C was 4·2% (95% CI: 3·0–5·7) and 0·7% (95% CI: 0·4–1·2), respectively. HIV and HBV coinfection was 0·5% (95% CI: 0·2–1·1), whereas HIV and HCV coinfection was 0·1% (95% CI: 0·0–0·5); no coinfection for all viruses was observed (Table 2).

**Table 2:** The prevalence of HIV, hepatitis B, hepatitis C and their coinfection among MSM, Rwanda, 2021

**Province**	**N: Unadjusted**	**HIV Prevalence** Adjusted % (95% CI)	**RITA Recent**^a^ Adjusted % (95% CI)	**Hepatitis B Prevalence** Adjusted % (95% CI)	**Hepatitis C Prevalence** Adjusted % (95% CI)	**HIV-Hepatitis B coinfection** Adjusted % (95% CI)	**HIV-Hepatitis C coinfection** Adjusted % (95% CI)
East	126	10·3 (5·4—18·7)	^c^	4·3 (1·4—11·9)	^b^	^c^	^c^
City of Kigali	1021	7·6 (5·7—10·1)	^c^	5·0 (3·3—7·5)	0·5 (0·2—1·3)	^c^	^c^
North	303	4·5 (2·3—8·6)	^c^	3·9 (1·6—8·9)	0·9 (0·2—4·2)	^c^	^c^
South	152	5·3 2·0—13·6)	^c^	2·5 (0·6—10·1)	0·5 (0·0—3·2)	^c^	^c^
West	609	5·0 (3·3—7·4)	^c^	3·2 (1·9—5·1)	1·8 (0·9—3·5)	^c^	^c^
Overall	**2,211**	**6·9 (5·5 -8·6)**	**12·9 (5·5—27·3)**	**4·2 (3·0—5·7)**	**0·7 (0·4—1·2)**	**0·5 (0·2- 1·1)**	**0·1 (0·0 -0·5)**

^a^Tested for only those identified to be HIV-positive

^b^No hepatitis C cases were identified

^c^Too small to report at this level

Table 3 shows the bivariate logistic regression model for individual factors’ association with HIV prevalence. The significant factors at the bivariable level include age, province, occupation, drug use, age at first anal sex with a man, circumcision, ever suffering any violence or abuse because of having sex with other men, involvement in commercial sex, condom use, and alcohol consumption frequency.

**Table 3:** Bivariate analysis of the association between HIV prevalence and behavioral and sociodemographic characteristics among MSM (*n* = 2,211), Rwanda, 2021

	*N* (Unweighted)	% HIV-Positive	95% CI	X2 (*p* value)
**Age group (a)**				
18–19	181	2·9	[1·0—8·0]	< 0·001**
20–24	840	3·0	[1·8—5·1]	
25–29	573	6·7	[4·3—10·3]	
30 +	615	15·0	[11·0—20·1]	
**Province**				
East	126	10·3	[5·4—18·7]	0·028**
Kigali	1,021	7·6	[5·7—10·1]	
North	303	4·5	[2·3—8·6]	
South	152	5·3	[2·0—13·6]	
West	609	5·0	[3·3—7·4]	
**Level of education completed (a)**				
No education/primary	962	7·6	[5·5—10·4]	*0·191*
Vocational/secondary/higher	1,157	5·7	[4·0—8·1]	
**Main occupation**				
Not employed	449	8·3	[5·3—12·6]	0·017**
Long-term regular work	422	8·5	[5·2—13·7]	
Students	206	1·2	[0·4—3·7]	
parttime staff/Casual laborer	549	7·3	[4·5—11·6]	
Other	585	6·7	[4·3—10·3]	
**Current marital status**				
Single	365	4·1	[2·3—7·1]	*0·210*
Ever married	1,846	7·3	[5·7—9·3]	
Refused to answer				
**Ever took drugs for any reason**	142	4·0	[1·5—10·3]	0·017**
Yes	2,004	7·1	[5·6—8·9]	
No	5	51·6	[9·4—91·7]	
**Ever took drugs just before having sex (a)**				
Yes	35	2·7	[0·6—12·6]	0·001**
No	2,175	6·9	[5·5—8·6]	
Refuse to answer	1	100·0		
**Ever injected drugs (a)**				
Yes	143	4·3	[1·5—11·4]	0·005**
No	832	7·3	[5·1—10·4]	
Refuse to answer	694	4·7	[3·0—7·3]	
**Age at first sexual intercourse with a man (a)**	251	8·8	[5·1—15·0]	
< 15	219	13·9	[7·7—23·9]	
15–18				
19–22	1,971	6·2	[4·8—7·9]	0·009**
23–25	238	12·4	[7·2—20·5]	
26 +	2	0·0		
**Circumcised**				
*Yes*	237	17·0	[11·1—25·0]	< 0·001**
*No*	1,972	5·7	[4·3—7·4]	
*Refuse to answer*	2	0·0		
**Ever suffer any violence or abuse because of having sex with other men**				
Yes	580	8·4	[5·5—12·6]	0·022**
No	1,555	5·9	[4·5—7·6]	
Refuse to answer	16	40·1	[14·6—72·4]	
**Ever been paid money, goods, or services for sex (a)**				
Yes	1,993	5·9	[4·6—7·5]	0·002**
No	217	15·5	[8·9—25·6]	
Refuse to answer	1	0·0		
**Condom use with all partners (general) (a)**				
Consistent	720	3·2	[1·8—5·6]	< 0·001**
Not consistent	1,404	9·4	[7·3—12·0]	
Refused to Answer	27	6·2	[0·9—33·4]	
**Frequency of alcohol consumption over the last 30 days (a)**				
Every day	168	6·3	[3·1—12·7]	0·004**
About every day	532	6·4	[3·8—10·5]	
At least once a week	769	6·2	[4·0—9·3]	
At least once a month	134	3·2	[1·3—7·5]	
None	11	30·6	[8·9—66·6]	
Do not know	3	0·0		

(a) Covariate frequencies may not add to total due to filters or missing responses

^**^ *p* < .05

In the final multivariable logistic regression full model, ever being 30 years and older (AOR: 5·16; 95% CI: 1·65 – 16·15), suffering for any type of violence or abuse because of having sex with other men (AOR: 3·42; 95% CI: 1·87 – 6·25), refusing to answer the question asking ever paid money, goods, or services for sex (AOR: 10·4; 95% CI: 3·30 – 32·84), inconsistently condom use (AOR: 3·15; 95% CI: 1·31 – 7·6) were all significantly associated with HIV infection after adjusting for known and available confounding factors (Table 4).

**Table 4:** Multivariable logistic regression analysis of risk factors associated with HIV prevalence among MSM, Rwanda, 2021

**Variable**	*N* (Unweighted)	**Crude OR**	**95% CI**		**Sign**	**AOR**	**95% CI**		**Sign**
**Age group**									
18–19	181	1·00	·	·		1·00	···	···	
20–24	840	1·05	0·31	3·53		1·01	0·30	3·43	
25–29	573	2·44	0·76	7·89		1·16	0·64	7·29	
30 +	615	5·98	1·93	18·57	**	5·16	1·65	16·15	**
**Province**									
East	126	1·00	·	·					
Kigali	1,021	0·72	0·33	1·53					
North	303	0·41	0·15	1·09					
South	152	0·49	0·14	1·69					
West	609	0·46	0·20	1·03					
**Main occupation**									
Not employed	449	1·00	·	·					
Long-term regular work	422	1·03	0·51	2·11					
Students	206	0·13	0·04	0·47					
parttime staff/Casual laborer	549	0·88	0·44	1·76					
Other	585	0·80	0·41	1·55					
**Ever took drugs for any reason**									
Yes	365	1·00	···	···					
No	1,846	1·87	0·97	3·61					
**Ever took drugs just before having sex**									
Yes	142	1·00	···	···					
No	2,004	1·83	0·65	5·18					
Refuse to answer	5	25·53	2·01	324·39	**				
**Ever injected drugs**									
Yes	35	1·00	···	···					
No	2,175	2·62	0·51	13·60					
Refuse to answer	1								
**Age at first sexual intercourse with a man**									
< 15	143	1·00	···	···					
15–18	832	1·78	0·58	5·48					
19–22	694	1·12	0·35	3·53					
23–25	251	2·18	0·65	7·36					
26 +	219	3·64	1·04	12·69					
**Circumcised**									
*Yes*	1,971	1·00	···	···					
*No*	238	2·15	1·11	4·14	**				
**Ever suffered any violence or abuse because of having sex with other men**									
No	237	1·00	···	···		1·00	···	···	
Yes	1,972	3·39	1·92	5·97	**	3·42	1·87	6·25	***
Refuse to answer	2					1·00	···	···	
**Ever been paid money, goods, or services for sex**									
No	580	1·00	···	···		1·00	···	···	
Yes	1,555	1·47	0·87	2·52		1·22	0·71	2·11	
Refuse to answer	16	10·7	2·66	43·33	**	10·4	3·30	32·84	***
**Condom use with all partners (general)**									
Consistent	720	1·00	···	···		1·00	···	···	
Not consistent	1,404	3·11	1·64	5·93	**	3·15	1·31	7·60	**
Refused to Answer	27	1·97	0·24	16·33		1·00			
**Frequency of alcohol consumption over the last 30 days**									
Every day	168	1·00	···	···		1·00	···	···	
About every day	532	1·01	0·40	2·56		1·08	0·42	2·75	
At least once a week	769	0·97	0·40	2·34		1·25	0·49	3·18	
At least once a month	134	0·49	0·15	1·58		0·47	0·12	1·78	
None	11	6·53	1·21	35·35	**	12·59	1·28	124·24	**
Do not know	3					1·00	···	···	

*OR* Odds Ratio, ***95%CI*** 95% confidence interval, ***AOR*** Adjusted Odds Ratio

^***^ *p* < ·01

^**^ *p* < ·05

## Discussion

The second integrated biological and behavioral survey among men who have sex with men in Rwanda in 2021 indicated that HIV prevalence among MSM in Rwanda (approximately one out of 14) is slightly over threefold that of men in the general population, with provincial differences and a substantial proportion of recent infections indicating ongoing transmission [18]. As indicated by the higher rates in the Eastern province and the City of Kigali, various factors, such as access to healthcare, education, and risk behaviors, may be prevalent in different provinces.

HBV shares the same mode of transmission as HIV, but it is highly contagious: 10 times more contagious than HCV and 100 times more contagious than HIV [33, 34]. The prevalence of HBV infection was slightly more than twice that of the general population. This disparity highlights the heightened vulnerability of MSM to HBV infection and indicates the need for targeted interventions to address this disparity effectively [35]. The low coinfection rates observed in this study highlight the possibility that HIV and viral hepatitis infection risk factors might be largely autonomous among the MSM population in Rwanda. However, the presence of coinfection cases underscores the importance of considering the syndemic nature of these infections and the need for comprehensive healthcare services for MSM.

The findings indicate a relatively low prevalence of HCV infection compared with the general population, which is encouraging, but it is essential to remain vigilant in addressing risk factors and transmission routes to prevent the further spread of these infections. HCV infection was found to be increasing with age, associated with unsterile injection equipment use, and multiple sexual partners [36, 37]. Therefore, the low prevalence of HCV observed might be attributed to the low level of intravenous drug use, being young (the majority are < 30 years old), and the low level of multiple sexual partners that characterize the MSM population in Rwanda.

Furthermore, 65·3% of Rwandans are younger than 30 years, and the study reveals that nearly half of the MSM population is younger than 25 years old, with an additional 25·7% falling in the age group of 25–29 years, indicating that a substantial proportion of MSM in Rwanda are young adults [38]. Younger age groups are often more susceptible to risky behaviors and may benefit from adopting tailored interventions to promote safer sexual practices and reduce the risk of HIV and other STIs [39]. Furthermore, the majority of MSM were single, and 46·8% had not gone beyond primary school in terms of education. Low educational attainment can be associated with limited knowledge about sexual health and prevention measures. Tailored educational campaigns may be necessary to increase awareness and promote safe sexual practices among this population [40].

The study also identifies a relatively high unemployment rate among MSM compared to 16·8% in the general population, which can impact access to healthcare and overall well-being [41]. Additionally, lifetime drug use among MSM was reported at 14·5%, with a small percentage using drugs before sex or having ever injected drugs. Substance use can increase the risk of engaging in risky sexual behaviors, highlighting the need for targeted interventions to address substance abuse and its association with HIV transmission [42].

Last, the finding that almost half of MSM had their first anal sex with men before the age of 18 raises concerns about early sexual debut and the potential for vulnerability to HIV and other STIs. However, findings indicate that slightly more than one-third of MSM reported consistent condom use for anal sex. This highlights the need for continued efforts to promote condom use as a critical preventive measure. Additionally, the majority of MSM reported being circumcised, with a median age of 12 years at circumcision. Male circumcision has been shown to reduce the risk of HIV transmission; in this study, 89·1% were circumcised, which is a positive finding in terms of prevention [43]. Additionally, the study findings indicated that uncircumcised MSM were two times more likely to be infected with HIV than circumcised MSM.

This study is subject to some limitations that warrant consideration when interpreting its findings. Firstly, the use of respondent-driven sampling (RDS) as the sampling method might have introduced uncontrolled biases and limitations to the generalizability of the results. RDS relies on the assumption of social network connectivity among participants, which may not always hold true and could lead to the underrepresentation of certain subgroups within the population. Secondly, the cross-sectional design of the study restricts its ability to establish causal relationships between variables. Furthermore, the study's inability to confirm active HCV infection among participants due to funding availability represents another limitation. Although individuals were screened for anti-HCV positivity, definitive confirmation of active infection through HCV RNA testing was not performed. However, it is noteworthy that the study considered referring anti-HCV positive screen participants for further testing, leveraging the availability of HCV testing services provided at no cost within the framework of HCV elimination in Rwanda.

## Conclusions

This study provides valuable insights into the prevalence and correlates of HIV and hepatitis infections among MSM in Rwanda. The findings underscore the importance of targeted interventions that address experiences of violence, promote condom use, scale-up PrEP, and engage more specifically with MSM involved in commercial sex to contribute to existing efforts in line with controlling the HIV epidemic. Furthermore, public health efforts may benefit from prioritizing comprehensive prevention strategies, including hepatitis B targeted vaccination, screening, and early diagnosis, by enhancing access to treatment and care to reduce the burden of hepatitis infections in this vulnerable population. By addressing the identified risk factors, Rwanda can take significant steps toward achieving better health outcomes and reducing HIV transmission among MSM.

## Data Availability

The data that informs the findings in this manuscript are client-level data fully owned by the Rwanda Ministry of Health and the Rwanda Biomedical Center (RBC). The data cannot be shared publicly to protect client privacy and confidentiality, considering that MSM are a sensitive key population. However, researchers who meet set criteria can access the data upon request and approval by RBC (data access requests are sent to: ‘info@rbc.gov.rw’).

## Abbreviations

AIC: Akaike Information Criterion
AIDS: Acquired Immunodeficiency Syndrome
AOR: Adjusted Odds Ratio
BBS: Bio-Behavioral Survey
CDC: US. Centers for Disease Control and Prevention
DGHT: Division of Global HIV and TB
FSW: Female Sex Workers
GHC: Global Health Center
HBsAg: Hepatitis B surface antigen
HBV: Hepatitis B virus
HCV: Hepatitis C virus
HIV: Human Immunodeficiency Virus
IQR: Interquartile Range
KPs: Key Populations
MSM: Men who have Sex with Men
ODK: Open Data Kit
OR: Odds Ratio
PEPFAR: The U.S. President’s Emergency Plan for AIDS Relief
PrEP: Pre-Exposure Prophylaxis
PSE: Population Size Estimates
PWID: People Who Inject Drugs
RBC: Rwanda Biomedical Centre
RDS: Respondent Driven Sampling
RTRI: Rapid Test for Recent Infection
RWF: Rwandan Francs
SSA: Sub-Saharan Africa
STIs: Sexually Transmitted Infections
TB: Tuberculosis
TG: Transgender people
UNAIDS: The Joint United Nations Programme on HIV/AIDS
USD: United States dollar
